# Polymorphisms in *TIM-3* and breast cancer susceptibility in Chinese women: A case-control study

**DOI:** 10.18632/oncotarget.9665

**Published:** 2016-05-27

**Authors:** Zheng Wang, Xinghan Liu, Xijing Wang, Tie Chong, Shuai Lin, Meng Wang, Xiaobin Ma, Kang Liu, Peng Xu, Yanjing Feng, Zhijun Dai

**Affiliations:** ^1^ Department of Oncology, Second Affiliated Hospital of Xi'an Jiaotong University, Xi'an 710004, China; ^2^ Department of Medical Oncology, Xi'an Central Hospital, Xi'an 710004, China; ^3^ Department of Urologic Surgery, Second Affiliated Hospital of Xi'an Jiaotong University, Xi'an 710004, China

**Keywords:** TIM-3, breast cancer, polymorphism, case-control study

## Abstract

Previous studies have found associations between polymorphisms in T cell immunoglobulin and mucin domain 3 (*TIM-3*) and increased risks of various cancers. However, the association between *TIM-3* polymorphisms and breast cancer (BC) remains uncertain. In this study, a total of 560 BC patients and 583 age, sex, and ethnicity-matched healthy controls from Northwest China were included. The polymorphisms were genotyped using Sequenom MassARRAY. The expression level of TIM-3 protein was detected by immunohistochemistry. We observed rs10053538 had a significantly increased risk of BC, comparing with the wild-type genotype even after Bonferroni correction. In addition, the rs4704853 G>A variants were more frequent among BC patients than the controls (GA + AA vs. GG: OR = 1.32, 95% CI = 1.03-1.69, P = 0.026); However, the significance was lost after Bonferroni correction (P = 0.078). Furthermore, rs10053538 was associated with lymph node metastasis. Age stratification revealed that among patients aged <49 years, those with the rs4704853 GA/AA genotype had a higher risk of BC; But there was no difference when Bonferroni correction was conducted. Immunohistochemical analysis showed that the expression of TIM-3 protein in the breast cancer tissues was higher in patients carrying the rs10053538 GT+TT genotype than those with GG genotype (P = 0.012). However, we failed to find any difference between BC patients and controls in any rs1036199 genetic model. These findings suggested that rs10053538 in *TIM-3* might increase susceptibility to BC and promote the progression of BC in Chinese women.

## INTRODUCTION

Cancer is one of the four leading causes of morbidity and mortality worldwide, with a high incidence in developing countries [1]. Breast cancer (BC) is the most common malignancy affecting women, with 1.8 million newly diagnosed cases in 2013, and is the leading contributor to disability-adjusted life-years [2–4]. The incidence of BC is increasing worldwide, as is the effect of hereditary BC. In Iceland, the number of carriers of mutations in the gene encoding breast cancer 2 (BRCA2) who are younger than 70 years was estimated to have increased fourfold (from 18.6% to 71.9%) over the last century, whereas the cumulative incidence of non-carriers also increased fourfold (from 2.6% to 10.7%) during the same period [5]. Women who test positive for familial *BRCA1/BRCA2* mutations are likely to have a higher risk of developing BC [6, 7]. Additionally, other factors such as chemokines, microRNAs, and single nucleotide polymorphisms (SNPs) have been proven to associate with BC risks, progression, and metastasis [8–10].

Proteins within the T-cell immunoglobulin and mucin domain (TIM) family, which comprises three members (*TIM-1*, *TIM-3* and *TIM-4*) encoded from genes on human chromosome 5q33.2, are expressed on T cells [11]. TIM-3, which negatively regulates T cell helper (Th1) cells and affects cytokine expression, has been associated with cancer susceptibility [12–14]. *TIM-3*, together with its ligand galectin-9, can induce T cell proliferation, cytotoxicity, and apoptosis [15, 16]. The primary involvement of *TIM-3*, as well as PD-1, in atherosclerosis, human immunodeficiency virus (HIV) infection, and regionally metastatic differentiated thyroid cancer contributes to exhaustion of T cells through pathways that regulate CD8^+^ T cells function [17–19].

To date, several lines of evidence have demonstrated associations of SNPs in *TIM-3* with increased risks of osteoarthritis, non-Hodgkin lymphoma, and non-small-cell lung cancer [20–22]. However, few articles have investigated the rs10053538, rs4704853, and rs1036199 polymorphisms, and none have estimated the associations between these three *TIM-3* SNPs and BC susceptibility. In this case-control study, we aimed to examine the associations of the *TIM-3* rs10053538, rs4704853, and rs1036199 polymorphisms with the risk of BC in a Northwest Chinese population.

## RESULTS

### Characteristics of the patients and controls

The characteristics of the 560 patients with BC and 583 controls are presented in Table 1. No significant differences were noted between patients with BC and controls in the distributions of age (P = 0.612) and menopausal status (P = 0.716). However, patients with BC and healthy controls differed significantly with respect to body mass index (BMI) (P = 0.038). Therefore, statistical analysis based on case-control comparisons was adjusted for age and BMI. The cases consisted of 313 (55.89%) estrogen receptor (ER) positive tumors, 305 (54.46%) progesterone receptor (PR)-positive tumors, 171 (30.54%) human epidermal growth factor receptor 2 (Her-2) positive tumors.

**Table 1:** Distributions of select variables in breast cancer cases and cancer-free controls

Characteristics		Cases	Control	*P* value ^*^
**Number**		560	583	
**Age (mean ± SD)**		49.09±11.02	48.80±8.28	0.612
**Menopausal status**				
**Premenopausal**		264	281	
**Postmenopausal**		296	302	0.716
**Body mass index(kg/m^2^)**				
**(mean ± SD)**		22.52±2.84	22.95±3.21	**0.038**
**Tumor size**	<2 cm	188		
	≥2 cm	372		
**LN metastasis**	Negative	236		
	Positive	324		
**ER**	Negative	247		
	Positive	313		
**PR**	Negative	255		
	Positive	305		
**Her-2**	Negative	389		
	Positive	171		

* T-test or two-sided *x*^2^-test.

LN, Axillary lymph node; ER, Estrogen receptor; PR, Progesterone receptor; HER-2, human epidermal growth factor receptor 2

### Association between *TIM-3* polymorphisms and breast cancer risk

All three SNPs of *TIM-3* gene were genotyped in 560 BC patients and 583 healthy control subjects, but some cases and controls were missing. The genotyping success rates of rs10053538, rs4704853, and rs1036199 were 99.83% (1141/1143), 99.56% (1138/1143), and 99.74% (1140/1143) respectively. The genotype and allelic frequencies of the *TIM-3* rs10053538, rs4704853, and rs1036199 polymorphisms are shown in Table 2. In the controls, the genotype frequencies of all three SNPs conformed to Hardy–Weinberg Equilibrium (HWE) (P = 0.10, 0.28, and 0.74 for rs10053538, rs4704853, and rs1036199, respectively), indicating that community genetic inheritance was balanced in the control samples and that these samples could represent the general population.

**Table 2:** Genotype and allele frequencies of the *TIM-3* polymorphisms among the cases and controls and the associations with breast cancer risk

Model	Genotype	Case(560)	Control(583)	OR (95% CI)^†^	P-value^*^	P_c_
**rs10053538**^a^						
**Codominant**	GG	173 (30.9%)	221 (38.0%)	1.00 (reference)		
	GT	313 (56.0%)	290 (49.8%)	1.38 (1.07-1.178)	**0.014**	**0.042**
	TT	73 (13.1%)	71 (12.2%)	1.31 (0.90-1.93)	0.162	NS
**Dominant**	GG	173 (30.9%)	221 (38.0%)	1.00 (reference)		
	GT+TT	386 (69.1%)	361 (62.0%)	1.37 (1.07-1.75)	**0.013**	**0.039**
**Recessive**	GG-GT	486 (86.9%)	511 (87.8%)	1.00 (reference)		
	TT	73 (13.1%)	71 (12.2%)	1.08 (0.76-1.53)	0.662	NS
**Allele**	G	659 (58.9%)	732 (62.9%)	1.00 (reference)		
	T	459 (41.1%)	432 (37.1%)	1.18 (0.997-1.40)	0.054	NS
**rs4704853^b^**						
**Codominant**	G/G	352 (63.1%)	402 (69.3%)	1.00 (reference)		
	G/A	191 (34.2%)	166 (28.6%)	1.31 (1.02-1.69)	**0.034**	NS
	A/A	15(2.7%)	12 (2.1%)	1.43 (0.66-3.09)	0.364	NS
**Dominant**	GG	352 (63.1%)	402 (69.3%)	1.00 (reference)		
	GA+AA	206 (36.9%)	178 (30.7%)	1.32 (1.03-1.69)	**0.026**	NS
**Recessive**	GG-GA	543 (97.3%)	568 (97.9%)	1.00 (reference)		
	AA	15 (2.7%)	12 (2.1%)	1.31 (0.61-2.82)	0.493	NS
**Allele**	G	895 (80.2%)	970 (83.6%)	1.00 (reference)		
	A	221 (19.8%)	190 (16.4%)	1.26 (1.02-1.56)	**0.034**	NS
**rs1036199^c^**						
**Codominant**	A/A	546 (97.7%)	565 (97.2%)	1.00 (reference)		
	C/A	13 (2.3%)	16 (2.8%)	0.84 (0.40-1.76)	0.646	NS
	C/C	0	0			
**Allele**	A	1105 (98.8%)	1146 (98.6%)	1.00 (reference)		
	C	13 (1.2%)	16 (1.4%)	0.84 (0.40-1.76)	0.648	NS

* Two-sided *x*^2^test for the distributions of genotype and allele frequencies.

† Adjusted for age and body mass index. P_c_, the Bonferroni correction of P values.

a Case missing n = 1; control missing n = 1;

b Case missing n = 2; control missing n = 3;

c Case missing n = 1; control missing n = 2.

Of all selected *TIM-3* SNPs, rs10053538 was identified as associated with the risk of BC. For rs10053538, we found that GT and GT + TT genotype carriers had a significantly increased risk of BC development (GT vs. GG: OR = 1.38, 95% CI = 1.07-1.78, P = 0.014, P_c_ = 0.042; GT + TT vs. GG: OR = 1.37, 95% CI = 1.07-1.75, P = 0.013, P_c_ = 0.039) relative to those with the GG genotype. For rs4704853 polymorphism, the GA and GA + AA frequencies among patients differed significantly from the GG genotype observed among the controls (GA vs. GG: OR = 1.31, 95% CI = 1.02-1.69, P = 0.034; GA + AA vs. GG: OR = 1.32, 95% CI = 1.03-1.69, P = 0.026); However, after Bonferroni correction, the significance was lost (GA vs. GG: P_c_ = 0.102; GA + AA vs. GG: P_c_ = 0.078). The differences in the G and A allele frequency distributions between patients and controls were also significant (OR = 1.261, 95% CI = 1.02-1.56, P = 0.034); But there was no significant difference when Bonferroni correction was performed (P_c_ = 0.102). We did not observe significant differences among the rs1036199 genotypes (CA vs. AA: OR = 0.84, 95% CI = 0.40-1.76, P = 0.646; C vs. A: OR = 0.84, 95% CI = 0.40-1.76, P = 0.648). We also obtained the statistical power of 0.81 and 0.83 for the two significant polymorphisms identified, rs10053538 and rs4704853, respectively. This showed that our sample size of 1143 was adequate and the study was sufficient to detect the associations of these two polymorphisms with BC risk.

### Association between *TIM-3* polymorphisms and clinical parameters in patients with breast cancer

To investigate potential associations between *TIM-3* polymorphisms and clinical features in patients with BC, we next analyzed the associations between these polymorphisms and a series of clinicopathological parameters, including tumor size, lymph node (LN) metastasis, ER, PR, and Her-2 statuses. As shown in Table 3, we identified a significantly higher frequency of the rs10053538 polymorphism among patients with a positive LN metastasis status (OR = 1.68, 95% CI = 1.17-2.42, P = 0.005, P_c_ = 0.025). Furthermore, individuals harboring the rs4704853 polymorphism were more likely to have a larger tumor size (≥2 cm) (OR = 1.57, 95% CI = 1.08-2.28, P = 0.018); The significance was lost after Bonferroni correction (P_c_ = 0.09). However, no significant associations were detected between the rs10053538 or rs4704853 polymorphism and other clinical parameters of in patients with BC.

**Table 3:** The associations between the *TIM-3* polymorphisms and clinical characteristics of breast cancer patients

Variables	rs10053538				rs4704853			
	GG (%)	GT+TT (%)	*P* ^*^	OR (95%CI)	GG (%)	GA+AA (%)	*P* ^*^	OR (95%CI)
**Tumor size**								
**<2 cm**	53 (28.3%)	134 (71.7%)		1.00 (reference)	130 (69.9%)	56 (30.1%)		1.00 (reference)
**≥2 cm**	120 (32.3%)	252 (67.7%)	0.345	0.83 (0.57-1.22)	222 (59.7%)	150 (40.3%)	**0.018**	1.57 (1.08-2.28)
**LN metastasis**								
**Negative**	88 (37.4%)	147 (62.6%)		1.00 (reference)	151 (64.0%)	85 (36.0%)		1.00 (reference)
**Positive**	85 (26.2%)	239 (73.8%)	**0.005**	1.68 (1.17-2.42)	201 (62.4%)	121 (37.6%)	0.706	1.07 (0.76-1.52)
**ER**								
**Negative**	74 (31.5%)	161 (68.5%)		1.00 (reference)	162 (65.9%)	84 (34.1%)		1.00 (reference)
**Positive**	99 (30.6%)	225 (69.4%)	0.814	1.05 (0.73-1.50)	190 (60.1%)	122 (39.1%)	0.228	1.24 (0.87-1.75)
**PR**								
**Negative**	70 (27.5%)	185 (72.5%)		1.00 (reference)	168 (66.4%)	85 (33.6%)		1.00 (reference)
**Positive**	103 (33.9%)	201 (66.1%)	0.101	0.74 (0.51-1.06)	184 (60.3%)	121 (39.7%)	0.139	1.30 (0.92-1.84)
**HER-2**								
**Negative**	118 (30.3%)	271 (69.7%)		1.00 (reference)	233 (52.2%)	156 (47.8%)		1.00 (reference)
**Positive**	55 (32.4%)	115 (67.6%)	0.635	0.91 (0.62-1.34)	119 (88.2%)	50 (11.8%)	**0.018**	0.63 (0.43-0.93)

* Two-sided *x*^2^ test for the distributions of genotype frequencies.

LN, Axillary lymph node; ER, Estrogen receptor; PR, Progesterone receptor; HER-2, human epidermal growth factor receptor 2.

### Stratified analysis of *TIM-3* polymorphisms and breast cancer risk

An age-stratified analysis of the effects of the *TIM-3* rs10053538 and rs4704853 polymorphisms on the risk of BC is presented in Table 4. The results indicated that the rs4704853 GA + AA genotype was significantly more frequent among young participants (OR = 1.49, 95% CI = 1.05-2.11, P = 0.025). But no difference was found when Bonferroni correction was conducted. No positive results were observed for the rs10053538 polymorphism.

**Table 4:** Stratified analyses on association between *TIM-3* polymorphisms and breast cancer risk

rs10053538					rs4704853				
Genotypes	Case (N= 559)	Control (N = 582)	P^*^	OR (95%CI)^†^	Genotypes	Case (N = 558)	Control (N = 580)	P^*^	OR (95%CI)^†^
	N (%)	N (%)				N (%)	N (%)		
**Age<49**					**Age<49**				
**GG**	89 (32.0%)	102 (38.5%)	0.114	1.00 (reference)	GG	170 (61.8%)	212 (70.7%)	**0.025**	1.00 (reference)
**GT+TT**	189 (68.0%)	163 (61.5%)		1.33 (0.93-1.89)	GA+AA	105 (38.2%)	88 (29.3%)		1.49 (1.05-2.11)
**Age≥49**					**Age≥49**				
**GG**	84 (29.9%)	119 (37.5%)	0.049	1.00 (reference)	GG	182 (64.3%)	190 (67.9%)	0.374	1.00 (reference)
**GT+TT**	197 (70.1%)	198 (62.5%)		1.41 (1.00-1.98)	GA+AA	101 (35.7%)	90 (32.1%)		1.17 (0.83-1.66)

* Two-sided *x*^2^ test for the distributions of genotype frequencies.

† Adjusted for age and age at menarche.

### *TIM-3* polymorphisms and TIM-3 protein expression

**Figure 1:**
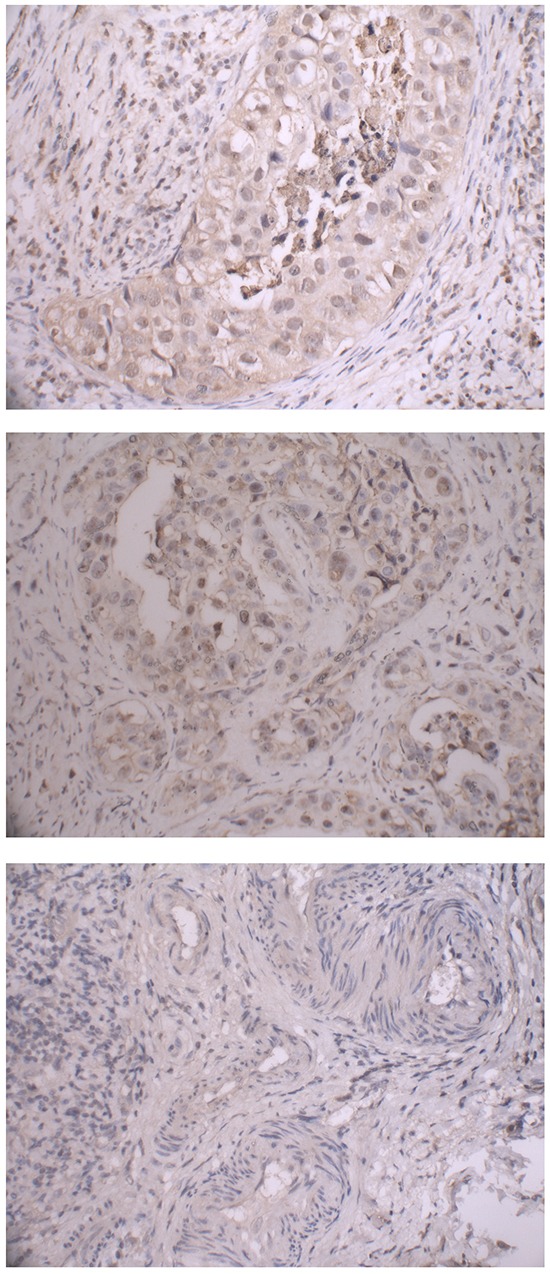
Immunohistochemical staining of TIM-3 protein in breast cancer tissues with rs10053538 GT, TT and GG genotypes Representive images were obtained at 100× magnification.

Immunohistochemistry (IHC) was performed in tumor specimens to quantify and localize the expression of TIM-3 protein, From the Figure 1, the brown-stained part of the immunohistochemical analysis picture is TIM-3 protein. Microscopy images showed both nuclear and cytoplasm localization of TIM-3. We evaluated the association between rs10053538 polymorphism and TIM-3 protein expression levels in 100 breast cancer tissues by IHC assay. Of these tissues, the frequency distribution of the GG, GT and TT genotypes was 28, 57 and 15, respectively. There was s significant difference in TIM-3 protein staining between the individuals with GT+TT and GG genotypes (average staining score: 3.7 versus 2.8 for GT+TT and GG genotypes, respectively, P = 0.012, Figure 1). Of the selected 100 tissues, the frequency distribution of the GG, GA and AA genotypes was 62, 37 and 1. Individuals carrying the GA+AA genotypes had no difference in TIM-3 protein staining score compared with individuals with GG genotype (average staining score: 3.0 versus 3.4 for GA+AA and GG genotypes, respectively, P = 0.104).

## DISCUSSION

Most cancers result from interactions between genes and the environment [23]. Recent studies have identified environmental factors as major contributors to human cancers, for which the risks are strongly genetically influenced [24]. Individuals using long-term immunosuppressive medications (azathioprine, cyclophosphamide, and cyclosporine) or those with underlying immunologic abnormalities, such as an autoimmune disease or viral infection, are particularly at risk of malignancy [25]. Immune dysregulation plays a vital role in both initiation and progression of autoimmune disease [26]. The evidence indicates that immune suppression contributes to cancer progression. According to the immune surveillance theory, innate immunity is responsible for the early detection and elimination of malignant cells [27]. T cells and regulatory CD4^+^ T cells (Tregs) utilize a number of molecular pathways such as the expression of inhibitory molecules (PD-1) to suppress a variety of physiological and pathological immune responses [28, 29].

*TIM-3*, which is expressed by a subset of activated CD4^+^ T cells, has been identified as a negative regulator of immune tolerance in autoimmune and alloimmune responses [30]. *TIM-3* is a transmembrane protein that selectively identifies Th1 cells not Th2 cells [31]. Carcinoembryonic antigen cell adhesion molecule 1, another well-known molecule negatively regulating cellular activation, is co-expressed and forms a heterodimer with *TIM-3* to regulate *TIM-3*-mediated tolerance and exhaustion [32, 33]. Song et al. [34] observed that Tregs could affect the prognosis of acute lung injury by upregulating *TIM-3* expression. *TIM-3* expression was also observed on Tregs in the peripheral blood of chronically hepatitis C virus-infected individuals, and this protein was shown to affect cell proliferation and apoptosis during HIV infection by altering the balance between Tregs and effector T cells [35].

Previous studies have suggested associations between *TIM-3* polymorphisms and some cancers and immune diseases. *TIM-3* −574G/T and +4259T/G were identified in patients with HIV-related non-Hodgkin lymphoma, non-small-cell lung cancer, and pancreatic cancer [20, 21, 36]. Rs11742259, rs10515746, rs35690726 and rs1036199 in TIM-3 were significantly associated with rheumatoid arthritis [37]. However, there were no previous reports of a relationship between *TIM-3* polymorphisms and the risk of BC. In our study, we observed that the *TIM-3* genotype variants rs10053538 and rs4704853, but not rs1036199, were associated with an increased risk of BC. Heon et al. [38] found that IL-15 induced cell proliferation and interferon-γ production by blocking *TIM-3* activity. Blocking of *TIM-3* may be the therapeutic by enhancing the Th 1 cytokines response, down-regulating the Th2 cytokines response, and reducing IgG/IgE production [39]. We also observed that the rs10053538 genotype variants were associated with a positive LN metastasis status. In immunohistochemical analysis, the results showed that individuals with rs10053538 GT+TT genotypes had a higher TIM-3 protein expression, which was in accordance with our result that *TIM-3* rs10053538 GT+TT genotypes had a higher BC risk. It is probably because *TIM-3* could negatively regulate the immune function of T cells and its higher protein expression level may suppress the immune response of T cells to tumors, which contributes to tumorigenesis. Our results demonstrated that *TIM-3* rs10053538 polymorphism might play a critical role in BC susceptibility.

Our study has some limitations. First, the sample size was inadequate for a stratified analysis and for analyzing associations in patients with mixed-type BC. Second, we did not investigate whether predisposing factors, including high-dose radiation exposure, alcohol consumption, and postmenopausal obesity, were associated with the risk of BC because of a lack of data from patients with BC and controls. In our future study, we will need to assess the effects of these factors on the risk of BC.

In conclusion, our case-control study indicates that rs10053538 GT+TT genetic variant in *TIM-3* had positive effects on BC susceptibility and progression in a population of Chinese women. Further functional studies and large population-based prospective studies will be required to further elucidate the influence of *TIM-3* polymorphisms on BC.

## MATERIALS AND METHODS

### Ethics statement

The study was approved by the Institutional Review Board of Xi'an Jiaotong University (Xi'an, China). Written informed consent was obtained from all participants involved in the study at the time of recruitment.

### Study participants

This case-control study included 560 patients with BC and 583 cancer-free controls, as we described previously [40, 41]. All participants were recruited without the restrictions of age. All patients were diagnosed with pathologically confirmed sporadic BC between January 2013 and October 2014 at the Second Affiliated Hospital of Xi'an Jiaotong University, China. Patients who received preoperative chemotherapy or radiotherapy or had a previous history of other types of cancer were excluded from the study. The controls were randomly selected from among healthy volunteers who underwent annual physical examinations in the hospital outpatient department and had no previous history of cancer. Controls were frequency matched to the patients according to age (±5 years). All methods were carried out in accordance with the approved guideline. All participants who provided written informed consent were interviewed to obtain detailed information about self-administration. After the interview, a venous blood sample (approximately 2 mL) was collected from each participant.

### DNA extraction

Whole blood samples were collected into EDTA-coated tubes and stored at −80°C after centrifugation until further analysis. Genomic DNA was extracted from whole blood using a standard phenol–chloroform extraction method. DNA concentrations were measured via spectrometry (DU530 UV/VIS spectrophotometer; Beckman Instruments, Fullerton, CA, USA).

### SNP selection and genotyping

For our study, we selected candidate SNPs in TIM according to HapMap data from a Chinese population. To achieve a power of at least 50%, only SNPs with a minor allele frequency (MAF) >0.01 was included. MassARRAY Assay Design 3.0 Software (Sequenom Laboratories, San Diego, CA, USA) was used to design a multiplexed SNP MassEXTEND assay. Finally, a total of three SNPs in TIM were included in this study. Genotyping of TIM-3 SNPs was performed with Sequenom MassARRAY RS1000, according to the manufacturer's instructions. The corresponding primers used for each SNP in this study are listed in Table 5. Sequenom Typer 3.0 Software was used for the data analyses.

**Table 5:** Primers used for this study

SNP_ID	1st-PCRP	2nd-PCRP	UEP_SEQ
**rs10053538**	ACGTTGGATGCGGTGGCTATGCCTGTAAAC	ACGTTGGATGCATGTTGGTCAGGCTGTTCT	AGGCGATCCACCCGCCTC
**rs4704853**	ACGTTGGATGTCATGCATACAAGGTGCCCC	ACGTTGGATGGGCTGGAACTCAACACTTTC	ATGGCCAAAGCCTCT
**rs1036199**	ACGTTGGATGCCTGGTGGTAAGCATCCTTG	ACGTTGGATGCTGACATTAGCCAAGGTCAC	gCCCCTGCACCGACTC

### Immunohistochemistry (IHC)

From the patient group, we selected 100 tissue specimens from October 2013 to October 2014 and performed immunohistochemical analysis of paraffin sections embedded breast cancer tissues. Firstly, parafin sections were roasted at 60°C for two hour, then deparaffinized and rehydrated through dimethylbenzene for 30 min and a descending alcohol series for 5 min, respectively. Endogenous peroxidase activity was blocked with 3% hydrogen peroxide for 20 minutes at room temperature. Then wash the slides with running water for 10 min before being exposed to the antigen retrieval system (0.01M sodium citrate, 0.05% Tween 20, pH6.0) in electromagnetic oven for 10min. Nonspecific stainings were blocked with closed serum for 15 min at room temperature. Then dump the excess liquid and dropped a primary antibody (1:100, ab185703, abcam, America) for incubation at 4°C overnight. After washing the slides with 0.01 mol/L phosphate buffer saline (PBS) for 3 times for 5min each time, incubated the slides with rabbit antibody (1:50, Boster, China) 30minutes at 37°C. Then rinsed the slides in PBS 3 times for 5min each time and stained the slides with DAB peroxidase substrate kit (Gene Tech, China). The slides were washed by running water for 10 min and conterstained with hematoxylin. The tissue sections then were observed under a microscope, after being dehydrated, cleared and finally mounted with neutral gum.

### Evaluation of the IHC variable

The TIM-3 expression was evaluated by two independent pathologists in a blind fashion. The stained sections were screened under low power (×100-fold magnification) to identify representative fields. TIM-3 positive cells were then countered under high power (×400-fold magnification) in 8 fields of vision and got the average. The staining results were calculated by multiplying the intensity and percentage of positive cells, and categorized as follows: no staining =0, weak staining =1, moderate staining =2, and strong staining =3. The percentage of the stained cells was categorized as follows: ≤ 5% =0, >5% but <25% =1, >25% but <50% =2, and >50% =3. Then the staining results were calculated with semi-quantitative analysis (HSCORE system = stain intensity ×the percentage of the stained cells). The score of 0-1 was consider as negative outcome (−), 2-3 weak positive (+), 4-6 moderate positive (++), >6 strong positive (+++).

### Statistical analysis

The statistical power of the case-control study was calculated using QUANTO software 1.2.4 (University of Southern California, Los Angeles, CA, USA). Hardy–Weinberg Equilibrium (HWE) was tested for each SNP before the analysis. The Student *t*-test or the χ^2^ test was used to compare differences in the distributions of demographic characteristics and selected variables, as well as the genotype frequency distributions between patients and controls. We conducted a case-control study for all of the subjects, and then the patients were stratified by clinical characteristics and age under the multivariate logistic regression model. Odds ratios (ORs) and 95% confidence intervals (CIs) were used to assessed the degree of association between the *TIM-3* rs10053538, rs4704853, and rs1036199 polymorphisms and BC. All statistical analyses were performed using SPSS 18.0 software for Windows (SPSS Inc., Chicago, IL, USA). Differences were considered statistically significant at a P_c_ < 0.05 after the p value was corrected by Bonferroni correction, and all statistical tests were two-sided.
